# Development of a deep learning system to detect glaucoma using macular vertical optical coherence tomography scans of myopic eyes

**DOI:** 10.1038/s41598-023-34794-5

**Published:** 2023-05-17

**Authors:** Ji-Ah Kim, Hanbit Yoon, Dayun Lee, MoonHyun Kim, JoonHee Choi, Eun Ji Lee, Tae-Woo Kim

**Affiliations:** 1 Department of Ophthalmology, Ewha Womans University College of Medicine, Ewha Womans University Seoul Hospital, Seoul, Korea; 2 Department of Machine Learning and Computer Vision, Sungkyunkwan University, Suwon, Korea; 3 Department of Computing, Sungkyunkwan University College of Computing and Informatics, Sungkyunkwan University, Suwon, Korea; 4 Hippo T&C, Suwon, Korea; 5 Samsung Semiconductor Inc., Suwon, Korea; 6 Department of Ophthalmology, Seoul National University College of Medicine, Seoul National University Bundang Hospital, 82, Gumi-ro, 173 Beon-gil, Bundang-gu, Seongnam, Gyeonggi-do, Seongnam, 23347 Korea

**Keywords:** Eye manifestations, Diagnostic markers

## Abstract

Myopia is one of the risk factors for glaucoma, making accurate diagnosis of glaucoma in myopic eyes particularly important. However, diagnosis of glaucoma in myopic eyes is challenging due to the frequent associations of distorted optic disc and distorted parapapillary and macular structures. Macular vertical scan has been suggested as a useful tool to detect glaucomatous retinal nerve fiber layer loss even in highly myopic eyes. The present study was performed to develop and validate a deep learning (DL) system to detect glaucoma in myopic eyes using macular vertical optical coherence tomography (OCT) scans and compare its diagnostic power with that of circumpapillary OCT scans. The study included a training set of 1416 eyes, a validation set of 471 eyes, a test set of 471 eyes, and an external test set of 249 eyes. The ability to diagnose glaucoma in eyes with large myopic parapapillary atrophy was greater with the vertical than the circumpapillary OCT scans, with areas under the receiver operating characteristic curves of 0.976 and 0.914, respectively. These findings suggest that DL artificial intelligence based on macular vertical scans may be a promising tool for diagnosis of glaucoma in myopic eyes.

## Introduction

Glaucoma is an irreversible eye disease responsible for approximately 12% of global blindness^1^. Myopia, particularly high myopia, has been shown to increase the risk of development of primary open angle glaucoma (POAG)^2–4^. Myopia affects approximately 1.6 billion people worldwide, with its prevalence being particularly high in East Asian populations^5^. The global prevalence of myopia is expected to further increase, affecting approximately 50% of the worldwide population by the year 2050^6^. Myopic glaucoma is therefore expected to become an important health issue in the future.

A tilted appearance and parapapillary atrophy (PPA) are hallmarks of the myopic optic disc^7,8^. Stretching of a posterior sclera induced by axial elongation results in a tilted disc appearance and a gamma zone, comprising the area external to the disc margin, with absence of both retinal pigment epithelium (RPE) and Bruch’s membrane.

Spectral-domain (SD) and swept-source optical coherence tomography (OCT) have become the most commonly used diagnostic tools to detect structural glaucomatous damage. OCT scans of circumpapillary retinal nerve fiber layer (RNFL) thickness are widely used to diagnose glaucoma. However, the circumpapillary RNFL thickness is not a useful parameter in highly myopic eyes because (1) myopic eyes have different circumpapillary RNFL profiles, (2) segmentation errors are often present in myopic eyes, and (3) scanning over a large area of PPA results in poor quality images. Macular cube scans may be used as a substitute, but they may also provide false information in highly myopic eyes with severe posterior scleral bowing. A tilted disc appearance, diffuse thinning of the RNFL and the presence of a tigroid fundus can also make the diagnosis of glaucoma difficult using conventional methods, such as stereo disc and red free fundus photographs.

Although single vertical line scans of the macula on SD-OCT images are symmetrical in healthy eyes^9,10^, this symmetry was not detected in glaucomatous eyes. One advantage of this method was that the images were not distorted even in highly myopic eyes having severe optic disc distortion or large areas of PPA^10^. This method may therefore be a useful diagnostic tool in highly myopic eyes. However, detecting asymmetry in macular images is subjective, with the accuracy affected by clinician experience.

Recent progress in artificial intelligence (AI) and the collection of large medical datasets have generated great interest in developing deep learning (DL) algorithms for diagnostic tests that can identify glaucoma lesions faster and more accurately than subjective evaluations and other traditional methods^11–14^. To date, however, few studies have evaluated these methods in highly myopic eyes, especially in extremely high myopic eyes with large areas of PPA.

The present study was designed to develop and validate a DL system that could detect glaucoma in myopic eyes using macular vertical OCT scans and to compare the diagnostic power of this system with the results of circumpapillary OCT scans.

## Results

The present study included 747 myopic healthy eyes and 1860 myopic glaucomatous eyes, including 136 healthy eyes and 224 glaucomatous eyes with a high degree of myopia and large PPA areas on circular OCT scans. These eyes were randomized to a training set, consisting of 393 myopic healthy eyes and 1023 myopic glaucomatous eyes; a validation set, consisting of 132 myopic healthy eyes and 339 myopic glaucomatous eyes; and a test set, consisting of 130 myopic healthy eyes and 341 myopic glaucomatous eyes. In addition, an external test dataset consisted of 92 myopic healthy eyes and 157 myopic glaucomatous eyes. Interobserver agreement regarding the diagnosis of glaucoma was excellent (κ = 0.959), as was interobserver agreement for eyes with large PPA areas involving the scanning circle (κ = 0.846).

Table 1 summarizes the clinical and demographic characteristics of the study participants. Comparisons of healthy and glaucomatous eyes in the training set showed that the percentage of male subjects was significantly higher in the glaucoma group (*p* < 0.001). In addition, axial length (AXL) was significantly longer (*p* = 0.002), RNFL significantly thinner (*p* < 0.001), and Humphrey visual field (HVF) mean deviation (MD) significantly lower (*p* < 0.001) in the glaucoma than in the control group. Similar results were observed in the validation and test datasets, except that AXL did not differ significantly in the glaucoma and control groups. Subjects with glaucoma in the external test dataset were significantly older (*p* = 0.012), consisted of a significantly higher percentage of male subjects (*p* = 0.040), and had significantly thinner RNFL (*p* < 0.001) and lower HVF MD (*p* < 0.001) than subjects in the control group.

**Table 1:** Clinical and demographic characteristics of subjects.

Variables	Training dataset		Validation dataset		Test dataset		External test dataset	
	Control (*n* = 393)	Glaucoma (*n* = 1023)	Control (*n* = 132)	Glaucoma (*n* = 339)	Control (*n* = 130)	Glaucoma (*n* = 341)	Control (*n* = 92)	Glaucoma (*n* = 157)
Age (years)	66.4 ± 12.4	67.3 ± 16.1	50.5 ± 13.8	51.5 ± 12.0	48.3 ± 10.9	49.6 ± 11.2	53.8 ± 15.5	58.6 ± 13.8
Male (%)	42.2	54.3	53.0	66.7	47.7	65.1	48.9	61.1
Axial length (mm)	24.84 ± 1.78	25.17 ± 1.75	27.74 ± 1.20	27.68 ± 1.30	27.59 ± 0.99	27.56 ± 0.95	25.32 ± 1.70	25.39 ± 1.88
RNFL thicknesses (µm)	93.0 ± 12.0	69.1 ± 14.8	90.0 ± 25.3	66.9 ± 14.3	88.3 ± 12.4	66.0 ± 18.5	94.4 ± 12.5	70.5 ± 16.2
HVF MD (dB)	− 1.80 ± 2.79	− 7.41 ± 7.53	− 2.02 ± 2.5	− 6.66 ± 6.40	− 2.26 ± 3.37	− 7.72 ± 7.31	− 2.22 ± 3.54	− 7.31 ± 6.64

Results are reported as mean ± standard deviation or n (%).

RNFL, retinal nerve fiber layer; HVF, Humphrey visual field; MD, mean deviation.

**Figure 1:**
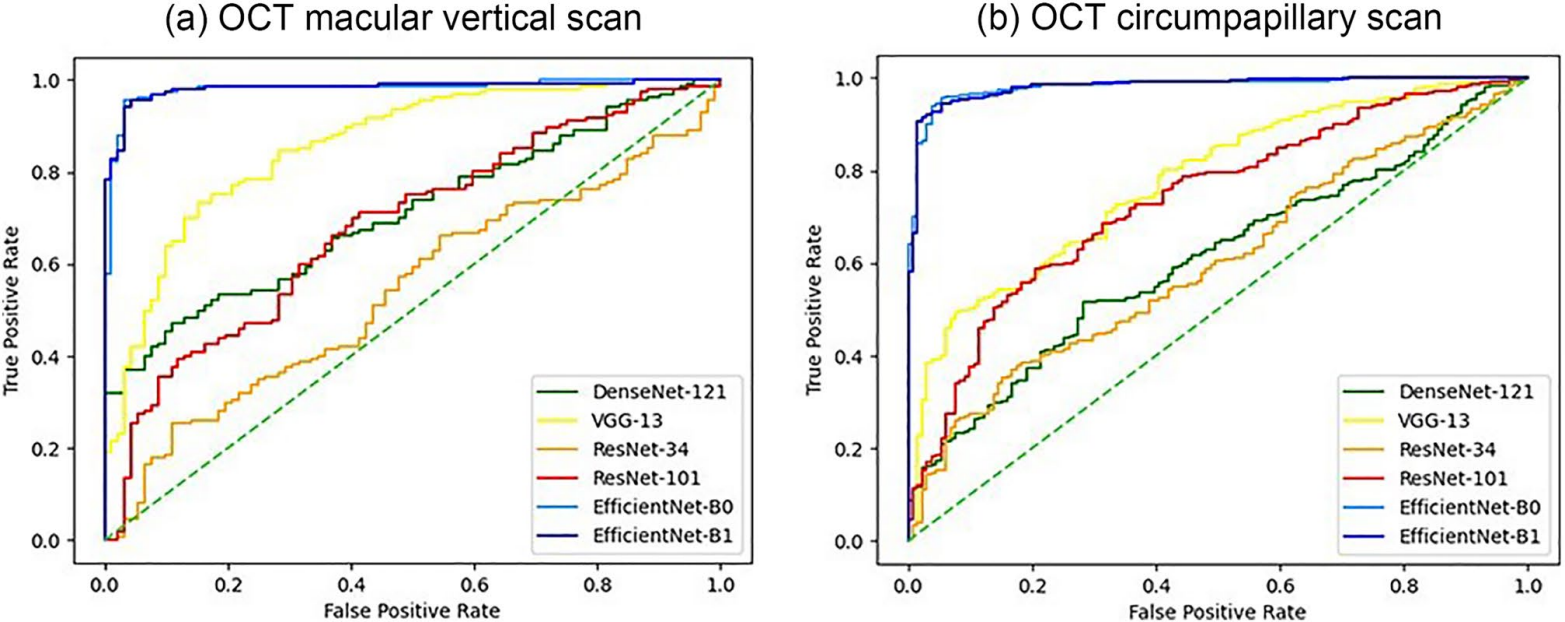
Receiver operating characteristic (ROC) curves summarizing the ability of the deep learning models to diagnose glaucoma in myopic patients. ROC curves of models using (**a**) macular vertical OCT scans and (**b**) circumpapillary OCT scans. EfficientNet models showed a better ability to diagnose myopic glaucoma than the other models using both macular vertical and circumpapillary OCT scans.

Table 2 shows the areas under the receiver operating characteristic curves (AUCs) for the probability of myopic glaucoma predicted by the DL algorithm in each model. The EfficientNet model showed superior performance compared with the other models (all *p* < 0.001, Fig. 1). There was no significant difference between EfficientNet-B1 and EfficientNet-B0 (*p* = 0.963). The AUCs of the DL system were higher using macular vertical OCT scans (0.981 for both the EfficientNet-B0 and B1 models) than using disc circumpapillary OCT scans (0.840 and 0.975, respectively; *p* = 0.002 for EfficientNet-B1). In the external test dataset, AUCs were also high using macular vertical OCT scans with EfficientNet models B0 (0.982) and B1 (0.984), which was not different from the result of internal test dataset (*p* = 0.813; Supplementary Table S1). The AUC was highest using macular vertical OCT scans with individual numeric data combination with the EfficientNet-B0 model (0.986, Table 2), similar to the result obtained using the external test dataset (AUC = 0.983).

**Table 2:** AUC results of the deep learning system using each model to diagnose myopic glaucoma in the internal test dataset, based on circumpapillary and macular vertical OCT scans and individual demographic and ophthalmic characteristics.

	AUC (95% Confidence interval)		
	Circumpapillary OCT	Macular vertical OCT	Macular vertical OCT and Patient characteristics*
DenseNet-121	0.627 (0.532–0.721)	0.610 (0.515–0.706)	0.949 (0.909–0.979)
VGG-13	0.671 (0.579–0.763)	0.776 (0.695–0.858)	0.912 (0.840–0.973)
ResNet-34	0.594 (0.498–0.690)	0.601 (0.505–0.697)	0.930 (0.885–0.970)
ResNet-101	0.741 (0.656–0.827)	0.738 (0.651–0.824)	0.938 (0.896–0.975)
EfficientNet-B0	0.840 (0.769–0.912)	0.981 (0.955–1.000)	0.986 (0.966–1.000)
EfficientNet-B1	0.975 (0.945–1.000)	0.981 (0.955–1.000)	0.995 (0.987–1.000)

AUC, area under the receiver operating characteristic curve; OCT, optical coherence tomography.

*Including axial length, mean deviation of Humphrey visual field tests, sex, and age.

**Figure 2:**
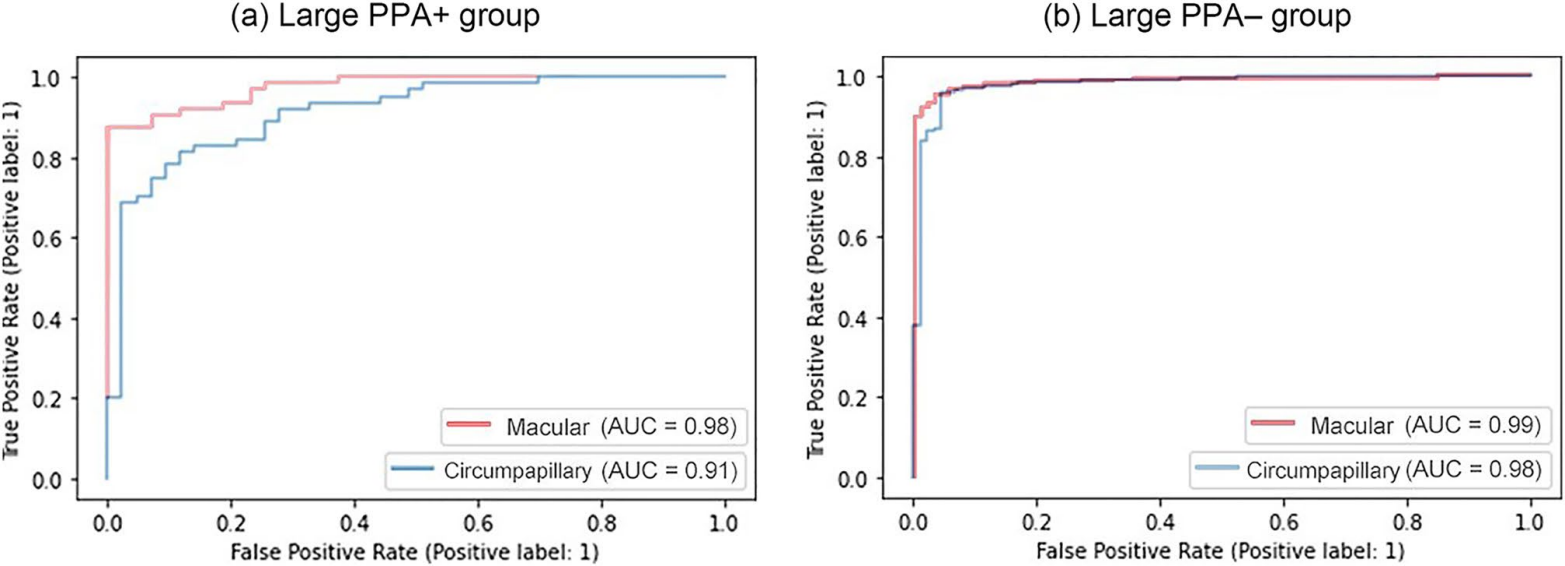
Receiver operating characteristic (ROC) curves in the test dataset, determined using the EfficientNet-B1 model for each group. *Red* and *blue* *lines* show the results from models using macular vertical and circumpapillary OCT scans, respectively. ROC curves of the (**a**) large PPA + group and (**b**) the large PPA– group. Macular vertical OCT scans showed better performance than circumpapillary OCT scans in the large PPA + group.

Subanalyses comparing the Large PPA + and Large PPA– groups found that AUCs for the probability predicted by the DL algorithm were higher using macular vertical OCT than circumpapillary OCT scan images (e.g., 0.976 vs. 0.914 for the large PPA + group, *p* = 0.003; Table 3). Figure 2 shows the AUC of each group based on the type of scan. Regardless of specificity, the model using macular vertical OCT scans showed greater sensitivity than the model using circumpapillary OCT scans for the large PPA + group (Fig. 2a), whereas the models showed similar curves for the large PPA– group (*p* = 0.082; Fig. 2b).

**Table 3:** Ability of the deep learning system using the EfficientNet-B1 model to diagnose myopic glaucoma in the internal validation dataset based on circumpapillary and vertical OCT scans in the large PPA + and large PPA– groups.

Variables	Large PPA + Group* (*n* = 360)			Large PPA– Group (*n* = 2247)		
	AUC (95% confidence interval)	Sensitivity at 90% specificity (%)	Sensitivity at 80% specificity (%)	AUC (95% confidenceinterval)	Sensitivity at 90% specificity (%)	Sensitivity at 80% specificity (%)
Circumpapillary OCT scan	0.914 (0.859–0.969)	75.0	82.8	0.981 (0.955﻿–1.008)	97.1	98.2
Vertical OCT scan	0.976 (0.946–1.006)	90.6	92.2	0.990 (0.970﻿–1.009)	97.1	98.6

OCT, optical coherence tomography; AUC, area under the receiver operating characteristic curve.

*Subjects classified by the presence of peripapillary atrophy overriding the 12 degree scan circle around the optic nerve on circumpapillary retinal nerve fiber layer OCT scans, leading to artifacts.

**Figure 3:**
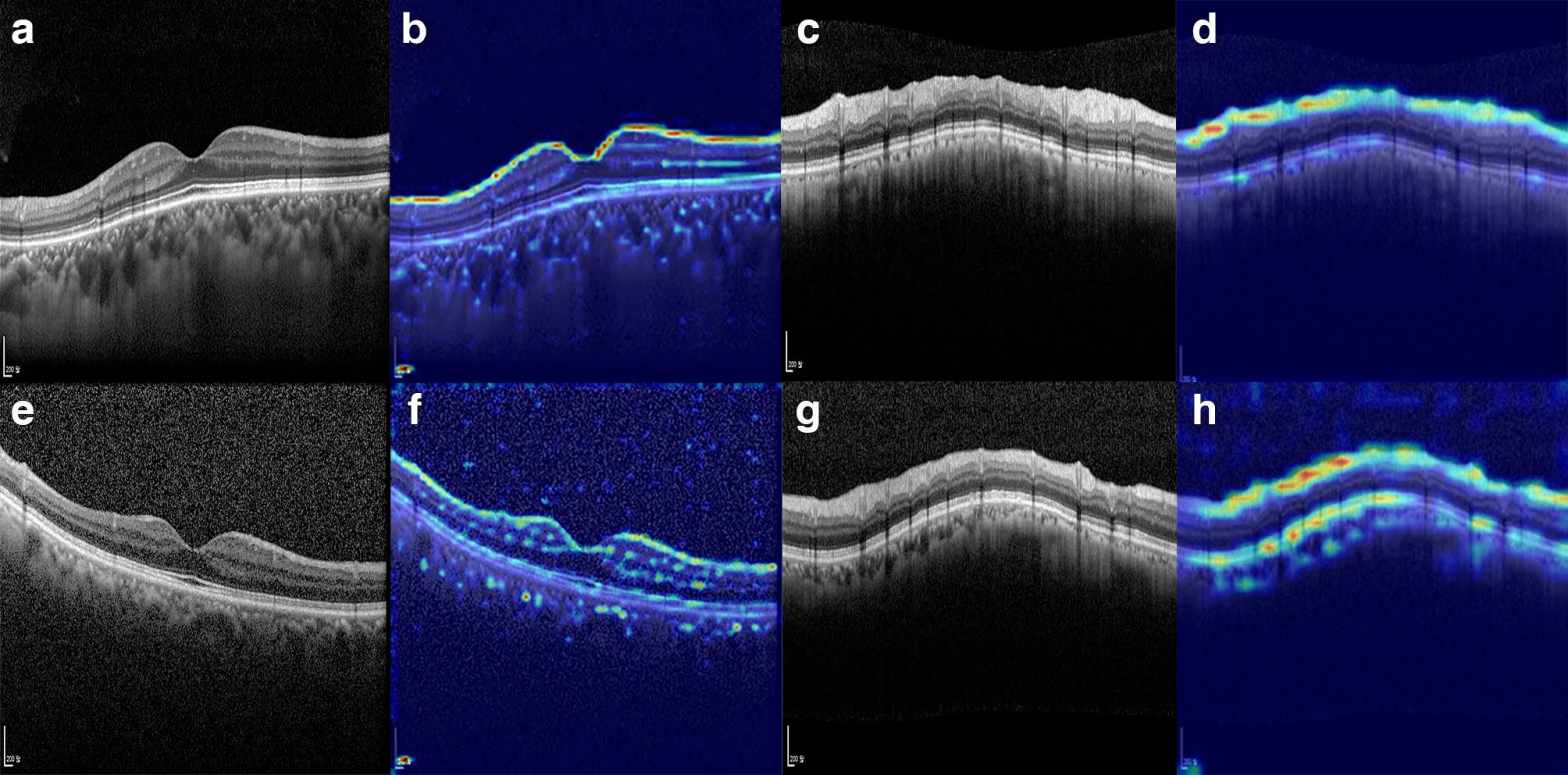
Heatmaps highlighting the regions of greatest weight in the deep learning algorithm classifications for (**a, b, c, d**) a healthy eye and (**e, f, g, h**) a glaucomatous eye. (**a, e**) Macular vertical spectral-domain optical coherence tomography (OCT) scans. (**b, f**) The same images as in panels a and c, respectively, after highlighting by the deep learning algorithm. (**c, g**) Circumpapillary spectral-domain OCT scans. (**d, h**) The same images as in panels c and g, respectively, after highlighting by the deep learning algorithm.

As a visual aid to help explain the results of the DL-based diagnosis system, heatmaps highlighting the important regions in each example are shown in Fig. 3. These findings confirmed that the heatmap for each dataset showed intensive activation of the RNFL on OCT scans.

## Discussion

The present study found that the convolutional neural network (CNN)-based DL model using EfficientNet-B1 architecture showed excellent performance in differentiating myopic eyes with and without glaucoma. Compared with the system using circumferential OCT scans, the system using macular vertical OCT scans showed a noninferior ability to detect glaucoma in myopic eyes. Similar results were observed in the external test dataset, which was not used in DL training. Furthermore, the system using macular vertical OCT scans showed higher diagnostic ability than the system using circumferential OCT scans in the large PPA + group, in which large areas of PPA overlap the circumpapillary OCT scan areas. The diagnostic ability of the system using macular vertical OCT scans was enhanced by the inclusion of demographic and ophthalmic parameters. To our knowledge, this is the first study to analyze the ability of an AI assisted system to diagnose glaucoma in highly myopic eyes with large PPA areas affecting circumpapillary OCT scans.

Many studies have sought to improve the ability of SD-OCT to diagnose glaucoma in highly myopic eyes. Methods showing good ability to distinguish between highly myopic eyes with and without glaucoma include measurements of macular ganglion cell-inner plexiform layer (GCIPL) thickness^15,16^ and its asymmetry^17^, optic disc rim measurements such as Bruch’s membrane opening minimum rim width (BMO-MRW) or 3-dimensional neuroretinal rim thickness^18,19^, wide-field map from swept-source OCT^20^, and OCT angiography^21^. Studies of these methods, however, did not include eyes with large PPA areas. Large PPA areas in the circumpapillary region can affect the scan circle, leading to image artifact or false measurement of the OCT RNFL thickness. Despite including eyes with large PPA areas, the present study showed that this method provided similar or even higher diagnostic power than in previous studies. The high diagnostic power of the DL system in the present study may be due to the use of an advanced AI system and minimal artifacts of the macular vertical OCT scans. Moreover, the DL algorithm was trained to analyze entire SD-OCT images, potentially providing more information associated with glaucomatous damage than individual SD-OCT parameters^22^.

The use of original raw OCT scan images, rather than layer segmented images or measurements of RNFL thickness, controlled for the effect of segmentation error frequently observed in eyes with large PPA areas. The diagnostic ability of the segmentation free DL algorithm in the large PPA + group was higher when using macular vertical than circumpapillary OCT scan images. This may have been due to the frequency of low quality circumpapillary OCT scan images in highly myopic eyes resulting from the effects of large PPA areas on the scan circle. In contrast, macular vertical images were rarely of low quality, except for mirror image artifacts in highly myopic eyes. The relatively poor RNFL scan quality of circumpapillary OCT scans would therefore result in less information for the diagnosis of glaucoma than macular vertical OCT scans.

The system that used demographic and ophthalmic parameters in addition to macular vertical OCT scans showed higher diagnostic ability than the system using macular vertical OCT scans alone. In contrast to the present study, which found that the inclusion of AXL affected the diagnostic performance of the DL system, a previous study reported that the inclusion of AXL did not affect diagnostic performance^23^. This difference may have been due to differences in subject inclusion criteria, as the present study included even extremely high myopic eyes with large PPA areas. Moreover, reduced RNFL thickness was observed in axially elongated eyes^24^. In addition, the present study included demographic and ophthalmic parameters, such as age, sex, and MD of HVF, which were not included in the previous study. RNFL thickness has been reported to decrease significantly with age^25^, and sex has been reported to affect circumpapillary RNFL thickness^26^.

**Figure 4:**
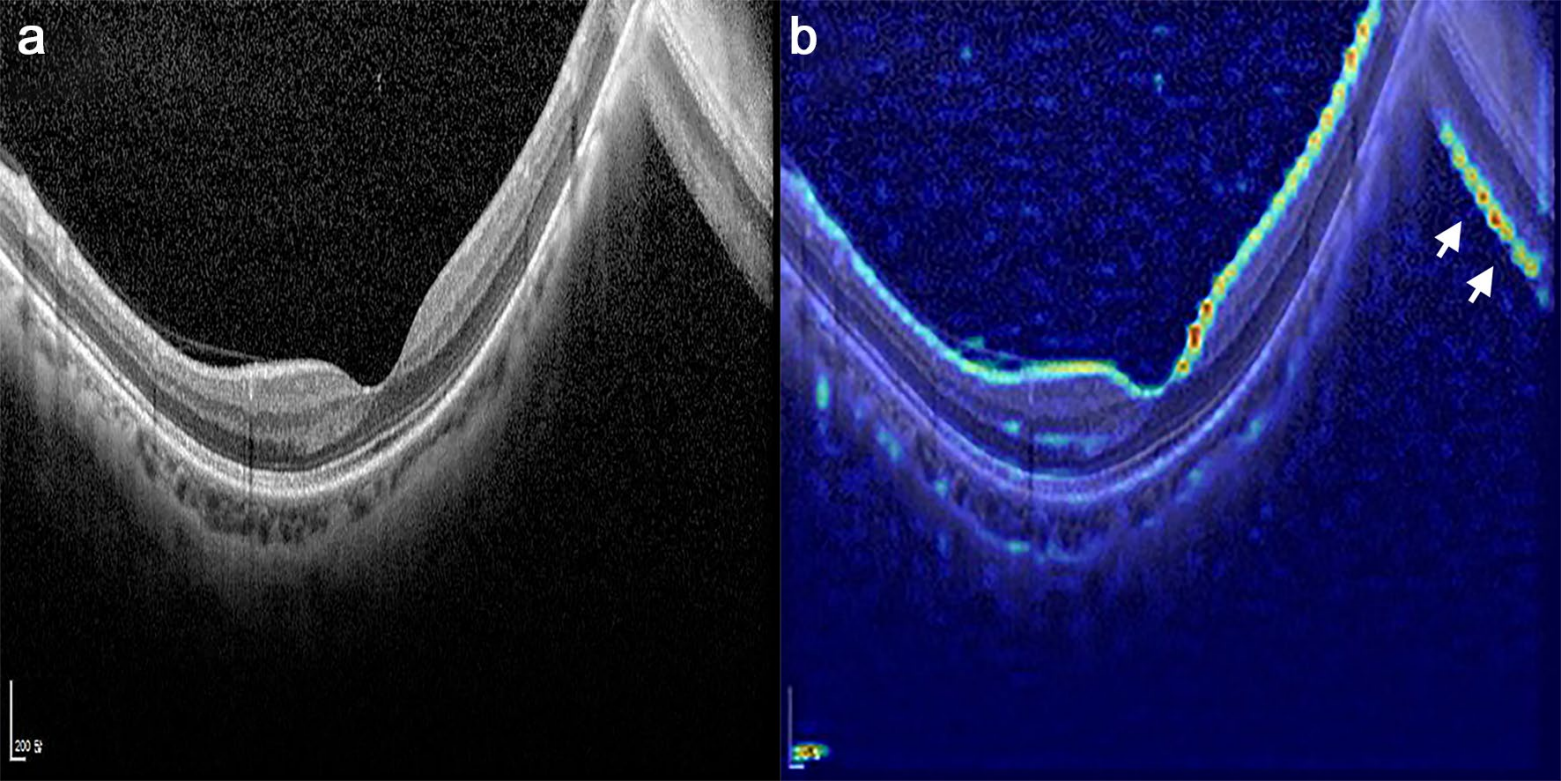
Example of a heatmap of a highly myopic eye with mirror-image artifact. (**a**) Macular vertical OCT scan. (**b**) The same image as in panel (**a**) after highlighting by the deep learning algorithm. Due to the highly bowed posterior pole, a cross-sectional image could not be fully captured in the scan window and the image over the frame is flipped. Although the image quality was lower in the mirror image than in the original image, the deep learning algorithm noticed the flipped image and highlighted the retinal nerve fiber layer of the image (*white arrows*).

The present study was robust in that OCT scans with low image quality were also included. OCT images of highly myopic, especially those with large PPA areas affecting the scan circle, are frequently of low quality. Poor image quality reduces the ability to diagnose glaucoma. In the present study, scans with low image quality were successfully classified by the DL algorithm, a finding confirmed by heatmap images. For example, the DL algorithm recognized flipped images and highlighted the RNFL of eyes with mirror-image artifacts, which are frequently present in highly myopic eyes (Fig. 4, Supplementary Fig. S1).

The present study had several limitations. First, the proportion of eyes in each group was not equal in our data set. The effects of this imbalance were therefore minimized by image augmentation^27^. Second, it was not possible to determine whether the decrease in RNFL thickness was due to the effects of myopia on the ganglion cell complex and/or optic nerve head, or whether glaucomatous damage was based on a single examination, indicating the need for longitudinal observations from an established baseline^28,29^. However, the ability of macular vertical scans to detect glaucomatous damage in myopic eyes has been verified. The present study assessed whether the DL algorithm could diagnose RNFL damage using vertical macular scans. Third, there could be biases inherent in the original images, which are further magnified with the expansion of the dataset. However, due to the nature of medical data, it is practically difficult to build a very large dataset such as a general image dataset, which makes the image data augmentation essential to improve performance. Fourth, ocular torsion is not uncommon occurrence in patients with amblyopia, and relying solely on vertical scans obtained by the Heidelberg Eye Explorer system may result in errors. Therefore, care should be taken to apply the current method in eyes with significant torsion. Fortunately, none of the patients in the present study had significant torsion or visual problem derived from ocular torsion. Fifth, we excluded eyes that were considered glaucoma suspects, as the diagnosis of glaucoma can be uncertain in highly myopic eyes. We included only patients who had clear evidence of glaucomatous damage and healthy eyes. The algorithm of this study cannot be applied to eyes that are considered glaucoma suspects, and further research is needed to address this issue. Sixth, the current method based on the hemifield asymmetry cannot be applied in far advanced glaucoma because asymmetry may not be a prominent feature if both superior and inferior hemifields are equally affected. However, in those cases, glaucoma detection is not difficult because of the total or nearly total absence of RNFL thickness. Seventh, there were gender inequalities between groups in the present study. According to Seo et al.^30^’s study on a total of 16.6 million people in South Korea, the prevalence of glaucoma was higher in males than in females. This may be one of the reasons for the gender imbalance between the glaucoma and healthy groups in the present study. We investigated whether there was gender-based differences in the AUC for glaucoma diagnosis. Our analysis using the EfficientNet-B1 model showed that the AUC was 0.984 for males and 0.978 for females, indicating high diagnostic performance for both genders. Finally, this study included only Korean subjects, which may limit the applicability of the present findings to other ethnic populations.

In conclusion, the DL system using EfficientNet on macular vertical scan showed good diagnostic ability to detect glaucomatous RNFL damage in myopic eyes including those with large PPA areas. The diagnostic ability of this system was further enhanced by including demographic and ophthalmic parameters. A DL algorithm based on using macular vertical scans may enable effective screening for glaucoma when clinicians trained to interpret OCT scans are not available. This system may also assist clinicians who diagnose glaucoma, particularly in highly myopic patients.

## Materials and methods

### Study subjects

This study included myopic subjects with and without POAG who visited Seoul National University Bundang Hospital and Ewha Seoul Hospital from January 2010 to August 2021. The study protocol was approved by the Institutional Review Boards (IRBs) of Seoul National University Bundang Hospital and Ewha Seoul Hospital and followed the tenets of the Declaration of Helsinki. Due to the retrospective nature of this study, patient informed consent was waived by the IRBs.

**Figure 5:**
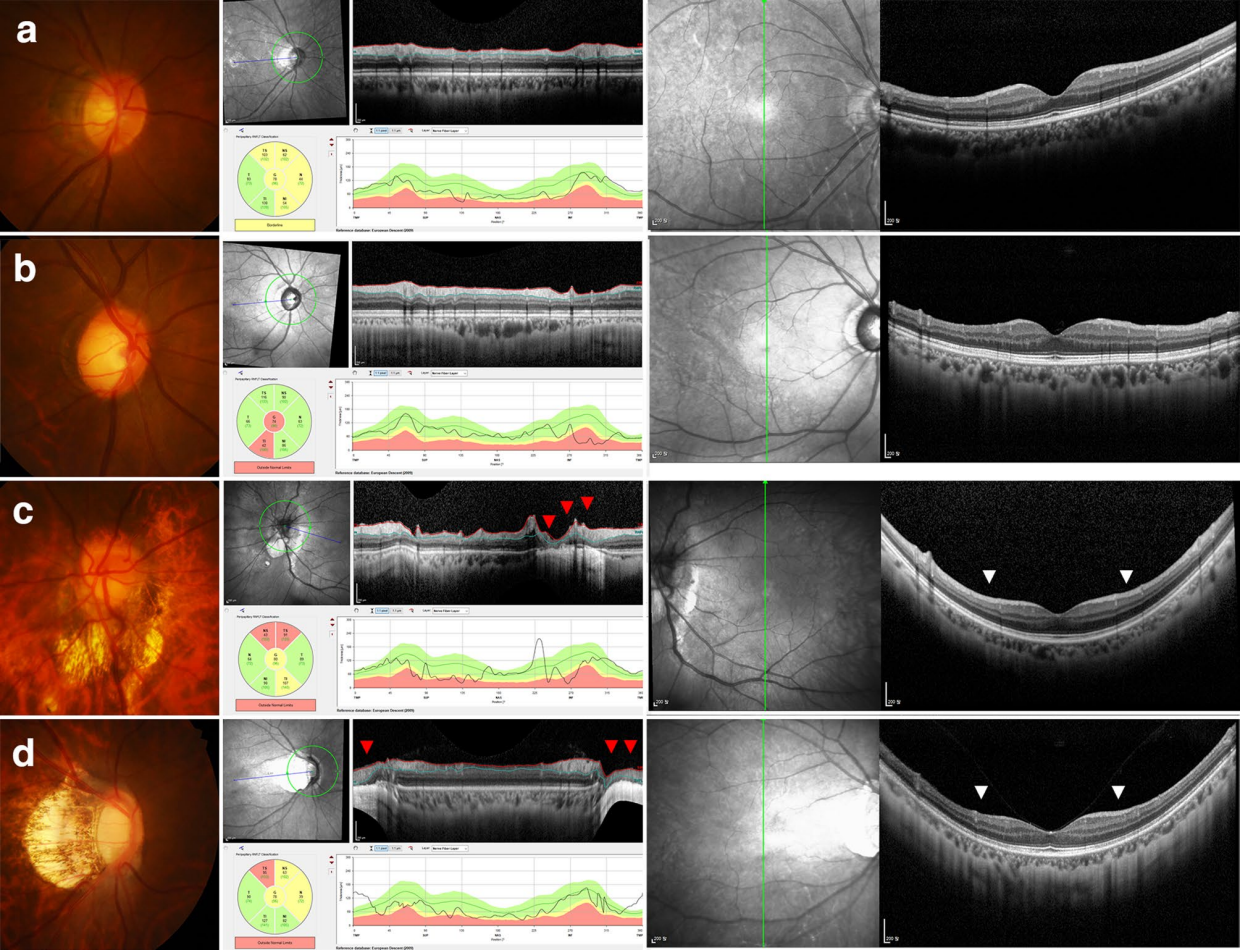
Representative myopic healthy and glaucomatous eyes. (**Left column**) Disc photography and (**Center column)** circumpapillary and (**Right column**) macular vertical OCT scans. (**a**) Healthy and (**b**) glaucomatous eyes without artifacts in circumpapillary OCT scans. (**c**) Healthy and (**d**) glaucomatous eyes with large peripapillary atrophy overriding the 12 degree scan circle around the optic nerve for OCT circumpapillary retinal nerve fiber layer scans. Due to image artifacts, segmentation errors were inevitable in circumpapillary OCT scans (*red arrowheads*). Symmetry and asymmetry are clearly visible (*white arrowheads*) on macular vertical scans.

Each subject underwent comprehensive ophthalmic examinations, including assessments of best-corrected visual acuity (VA), Goldmann applanation tonometry tests, refraction tests, slit-lamp biomicroscopy, gonioscopy, and fundus photography (Kowa VX-10, Kowa Medicals, Torrance, CA, USA). Other ophthalmic examinations included scanning of the circumpapillary RNFL and vertical macula using SD-OCT (Spectralis, Heidelberg Engineering, Heidelberg, Germany), and measurements of corneal curvature (KR-1800, Topcon, Tokyo, Japan), central corneal thickness (Orbscan II, Bausch & Lomb Surgical, Rochester, NY, USA), AXL (IOLMaster version 5, Carl Zeiss Meditec), and standard automated perimetry (Humphrey Field Analyzer II 750, 24-2 Swedish interactive threshold algorithm, Carl Zeiss Meditec, Dublin, CA, USA). Subjects were classified by the presence or absence of PPA areas overriding the 12 degree scan circle around the optic nerve on circumpapillary RNFL scans, leading to artifacts that make glaucoma diagnosis difficult (Large PPA + group; Fig. 5).

POAG was defined as the presence of glaucomatous optic nerve damage (i.e., NRR thinning/notching and an RNFL defect in the corresponding region on red-free fundus photography), asymmetric RNFL layer across the horizontal meridian on macular vertical OCT scan images^9,10^, a corresponding glaucomatous VF defect, an open iridocorneal angle on gonioscopic examination, no prior history of long-term use of steroid medications and no identifiable secondary cause of glaucoma. To assure reliable VF findings, the false-positive and false-negative rates had to be < 15%, and the rate of fixation losses had to be < 20%. Healthy myopic subjects had an IOP of < 21 mmHg without a history of increased IOP, an absence of a glaucomatous disc appearance, no visible RNFL defect on red-free fundus photography, and a symmetric RNFL layer across the horizontal meridian on macular vertical OCT scan images. VF defects due to myopia were defined as VF changes that were not glaucomatous and could not be explained by a disc photograph or RNFL distribution. Some eyes showed an enlarged blind spot associated with large PPA areas, a vertical step, or partial peripheral rim observed in highly myopic eyes^31,32^. Myopic eyes with AXL ≥ 23.0 mm were included^33,34^. Eyes were excluded if they had a history of intraocular surgery, except for uneventful cataract surgery; any retinal disease, such as diabetic retinopathy, epiretinal membrane, or retinoschisis; or a neurological disease such as a pituitary tumor.

Each eye was diagnosed as glaucomatous or non-glaucomatous by two experienced glaucoma specialists (T.-W.K. and J.-A.K.) independently. Diagnoses were based on observation of disc and RNFL photographs, circumpapillary and macular vertical OCT scans^10^, and VFs, without consideration of the clinical characteristics of the subjects. The symmetry of RNFL appearance in the superior and inferior hemispheres was evaluated on macular vertical OCT scans (Fig. 5). Eyes were classified as healthy (normal appearance of the macular ganglion cell layer and RNFL with symmetry in the two hemispheres) and glaucomatous (asymmetrical or diminished macular ganglion cell layer and RNFL). Disagreements between the two observers were resolved by consensus.

### Spectral-domain optical coherence tomography

Each SD-OCT examination included macular vertical and circumpapillary RNFL scans. The Spectralis OCT system produces clear retinal layer images, in which the ganglion cell layer and RNFL boundaries are clearly distinguishable. The Spectralis OCT includes an automatic real-time (ART) function, which utilizes TruTrack™ image alignment software (i.e., the eye tracking system). Following ART activation, multiple frames of the same scanning location can be obtained during the scanning process, with images averaged for speckle noise reduction. On circumpapillary RNFL scans, the scan circle around the optic nerve consisted of 768 A-scans 12 degrees in diameter. Each macular vertical B-scan spanned 30 degrees and consisted of 768 A-scans. Potential magnification errors were avoided by entering the corneal curvature of each eye into the Spectralis OCT system before scanning. OCT scans with low image quality were included. Not only the low quality score presented by the Heidelberg OCT viewer program, but also the images showing flipping or having large PPA that affects the scan circle of OCT were graded as the low image quality scans.

### Datasets

Myopic subjects with and without glaucoma who visited the glaucoma clinic of Seoul National University Bundang Hospital were divided into training, internal validation, and test sets by random sampling. The training set consisted of macular vertical and disc circumpapillary OCT images without segmentation of 1023 eyes of 698 patients with POAG and 393 eyes of 309 healthy subjects, for a total of 2832 OCT training images. OCT images were similarly acquired for the internal and external validation datasets and for test sets. The internal validation dataset consisted of images of 339 eyes of 253 patients with POAG and 132 eyes of 107 healthy subjects, for a total of 942 OCT internal validation images. The test dataset consisted of images of 341 eyes of 229 patients with POAG and 130 eyes of 102 healthy subjects, for a total of 942 OCT test images. The external test set, consisting of images of eyes from subjects at Ewha Womans University Medical Center, included images of 157 eyes of 118 patients with POAG and 92 eyes of 71 healthy subjects, for a total of 498 OCT external test images.

### Image augmentation

Heidelberg Eye Explorer (software version 1.10.4.0, Heidelberg Engineering), a viewer program provided with the Spectralis OCT device, provides the raw OCT scan results with en-face infrared reflectance image. These images were manually cropped to exclude unnecessary areas other than the regions of interest. The pixel values of the cropped images were scaled to a range of 0–1 and converted to a resolution of 260 × 260.

To improve the performance of the model, the training set was augmented using four image transformations: rotation, flip, blur, and unsharp mask. Data augmentation was required, especially when the training data set was small, considering the number of DL parameters, to avoid overfitting. Blurring and unsharp mask operations during image processing controlled pixel contrast along the boundaries of the object. In this study, these operations were applied to original OCT images to provide the DL system with variations in RNFL boundaries, which is a key element in determining RNFL thickness. During this process, the model becomes robust relative to noise at RNFL boundaries, enabling the model to extract only important information. Blurring was performed by weighted averaging with neighboring pixels using a Gaussian function to make boundaries less sharp. In contrast, unsharp mask operations were utilized to increase image sharpness, thereby enhancing RNFL layer boundaries. Unsharp mask operations were performed by increasing contrast with respect to neighboring pixels in proportion to the difference between each pixel and the weighted average of neighboring pixels. Supplementary Fig. S2 shows an original training image and images augmented by blurring and unsharp mask operations. The RNFL boundary in Supplementary Fig. S2a was smoothed, whereas the boundary of Supplementary Fig. S2b was sharpened and the details in the image enhanced.

To train the system on differences between left and right eyes and tilting images, data augmentation included flipping and rotation of the images using the pillow library. Data augmentation in normal eyes included one y-axis flip and six rotations, ranging from – 15° and 15° in 5° increments, whereas data augmentation in eyes with glaucoma included two rotations (– 15°, 15°) and one y-axis flip. Data augmentation mitigated imbalances during the process of data aggregation. Before data augmentation, the ratio of OCT imaging data between eyes with and without glaucoma was 3:1, but this ratio was reduced to 1.2:1 following data augmentation.

### Deep learning architecture

CNN-based frameworks, such as DenseNet19^35^, VGG20^36^, ResNet21^37^, and EfficientNet22^38^, were evaluated for image classification These models were trained with a sizeable ImageNet data set. The VGG model replaced the 5 × 5 filter several times with a smaller 3 × 3 filter, increasing the nonlinearity of the model and reducing the number of parameters to be learned. Thus, the VGG reduced the computational volume while improving the model’s performance. ResNet utilizes residual learning to prevent gradient vanishing or gradient exploding problems when the layers get more profound, resulting in reduced performance. Residual learning, the most significant structural feature of ResNet, includes a shortcut connection that adds input to output values, allowing ResNet to build neural networks that are deeper than existing models while preventing gradient vanishing. DenseNet (Densely Connected Convolutional Networks) is a model that connects the feature maps of all layers to the feature maps of every next layer. It is similar to ResNet, except that it concatenates rather than adding feature maps. Unlike other CNN networks, all layers were directly connected to the next layer, allowing the features of the first layer to be transferred to the last layer. This enables feature reuse and solves the vanishing-gradient problem. This model uses fewer parameters than ResNet but shows higher performance. The baseline model of EfficientNet, EfficientNet B0, was developed by searching neural network architecture to optimize accuracy and efficiency (FLOPS). A compound scaling method, which adjusts the model’s depth, width, and resolution using a ‘compound coefficient’, was used to find a group of EfficientNets.

### Deep learning experiments

A model that classifies circumpapillary OCT images was designed. Because raw images include both fundus and circumpapillary images, the fundus images were removed by cropping the raw data to extract circumpapillary OCT images. Each training image was augmented using the blur, flip, rotate, and unsharp mask methods, and the circumpapillary OCT images were input into pre-trained VGG, ResNet, DenseNet, and EfficientNet to yield a glaucoma classification model. Based on the input size of the pretrained model, the images were resized to 3 × 260 × 260. The output of the last linear layer of each model was changed to 2 for binary classification. Batch size was set at 32, learning rate at 0.001, the optimizer as Adam^39^ and the epoch as 100.

The same method was used to classify vertical macular image data. Data in the training set were augmented in the same way as the circumpapillary images, with the training, validation, and test sets divided similarly. Each training, validation, and test dataset included a vertical macular image and a circumpapillary OCT image of the same person. Each DL model was trained using the same conditions, such as batch size, learning rate, optimizer, and epoch. After training, the ability of each CNN model to detect glaucoma was measured and the two types of OCT images compared. Performance was measured for both the SNUBH and EUMC datasets. The EUMC dataset was not involved in training, but was used as an external test set to assess the generalizability of the trained model. In addition, the test set was divided into two subgroups: the large PPA + and large PPA– groups. The ability of each CNN model to detect glaucoma in each subgroup was measured and compared according to the types of input images.

### Combined model with numeric properties

Individual patient demographic and ophthalmic characteristics, including age, sex, AXL, and MD, were also included for further analyses. This combined machine learning model was designed to detect glaucoma based not only on OCT images but also on these properties. The combined model consisted of two separate machine learning models, < D,A > , which were connected sequentially (Supplementary  Fig. S3). The first model, D, was a trained DL model to classify OCT images, whereas the second model, A, was another classification model which follows the model D to classify whole user data. For each trained deep learning model D, a corresponding second model, A, was developed. The trained DL model D for OCT images determined the posterior probability for glaucoma. This probability was input into model A, which also included the numeric demographic and ophthalmic properties of each subject as extra input features. Model A was developed using the AutoML (automated machine learning) tool [AutoML-healthcare] in supervised learning mode.

AutoML has been used in automatic data transformation and to select and parameterize machine learning models to maximize classification performance. One of these AutoML systems, Tree-based Pipeline Optimization Tool (TPOT)^40,41^, depicts the entire supervised machine learning process as a tree-based pipeline. Feature preprocessing, selection, and construction, along with model selection and parameter optimization, are represented as tree nodes. A genetic algorithm was utilized as a stochastic optimization algorithm to identify an optimized pipeline. In each generation, trees were randomly modified using mutation and crossover genetic operators. The fitness of each generated tree was measured and the optimal tree selected to construct a population set, which was transferred for the next round. Generation was set at 100, population at 100, and cross-validation at 10, followed by the creation of an optimized model in the experimental process.

In this study, AutoML was applied only to the SNUBH test dataset of 471 eyes, consisting of 131 normal eyes and 340 eyes with glaucoma. This dataset was randomized in a 2:8 ratio, consisting of 95 normal eyes and 376 eyes with glaucoma. TPOT uses machine learning models implemented in Scikit-learn, a general-purpose Python machine learning library, and optimizes algorithms using genetic programming methods. Accuracy was set as an objective function and the most suitable model for the current test set generated through TPOT.

### Data analysis

Except where stated otherwise, data are presented as means ± standard deviations. Interobserver agreements for the diagnosis of glaucoma were evaluated using kappa statistics (i.e., κ value). Continuous variables were compared using *t*-tests and categorical variables using χ^2^ tests. The diagnostic performance of the trained DL model was tested with independent datasets (i.e., the internal and external validation datasets), and the AUCs and 95% CIs were calculated. The DeLong test was used to test the statistical significance of the diagnostic performance difference between any two parameters^42^. All statistical analyses were performed using the Statistical Package for the Social Sciences (version 22.0, SPSS, Chicago, IL, USA), with *p* < 0.05 defined as statistically significant.

## Supplementary Information

Supplementary Information.

## Data Availability

The datasets generated during and/or analysed during the current study are available from the corresponding author on reasonable request.
