# Supplementary material for: Development of a deep learning system to detect glaucoma using macular vertical optical coherence tomography scans of myopic eyes
Source: Sci Rep. 2023 May 17;13:8040. doi: 10.1038/s41598-023-34794-5 (PMC10192443; doi:10.1038/s41598-023-34794-5)
Supplement: Supplementary file 1 — Supplementary Information. [file 41598_2023_34794_MOESM1_ESM.pdf]

Development of a Deep Learning System to Detect Glaucoma Using Macular Vertical Optical Coherence Tomography Scans of Myopic Eyes

Ji-Ah Kim[1,*], Hanbit Yoon[2,*], Dayun Lee[3], MoonHyun Kim[3,4], JoonHee Choi[5], Eun Ji Lee[6], Tae-Woo Kim[6]

[1]Department of Ophthalmology, Ewha Womans University College of Medicine, Ewha Womans University Seoul Hospital, Seoul, Korea

[2]Department of Machine Learning and Computer Vision, Sungkyunkwan University, Suwon, Korea

[3]Department of Computing, Sungkyunkwan University College of Computing and Informatics, Sungkyunkwan University, Suwon, Korea

[4]Hippo T&C, Suwon, Korea

[5]Samsung Semiconductor Inc., Korea

[6]Department of Ophthalmology, Seoul National University College of Medicine, Seoul National University Bundang Hospital, Seongnam, Korea

Supplementary Files

Supplementary Figure 1. Examples of the DL algorithm recognizing flipped images and highlighting the retinal nerve fiber layer of eyes with mirror-image artifacts.

Supplementary Figure 2. Examples of augmentation of blur and unsharp mask phases

Supplementary Figure 3. Combined model with baseline demographic and ophthalmic characteristics.

Supplementary Table 1. AUC results of the deep learning system using each model to diagnose myopic glaucoma in the external test dataset, based on circumpapillary and macular vertical OCT scans.

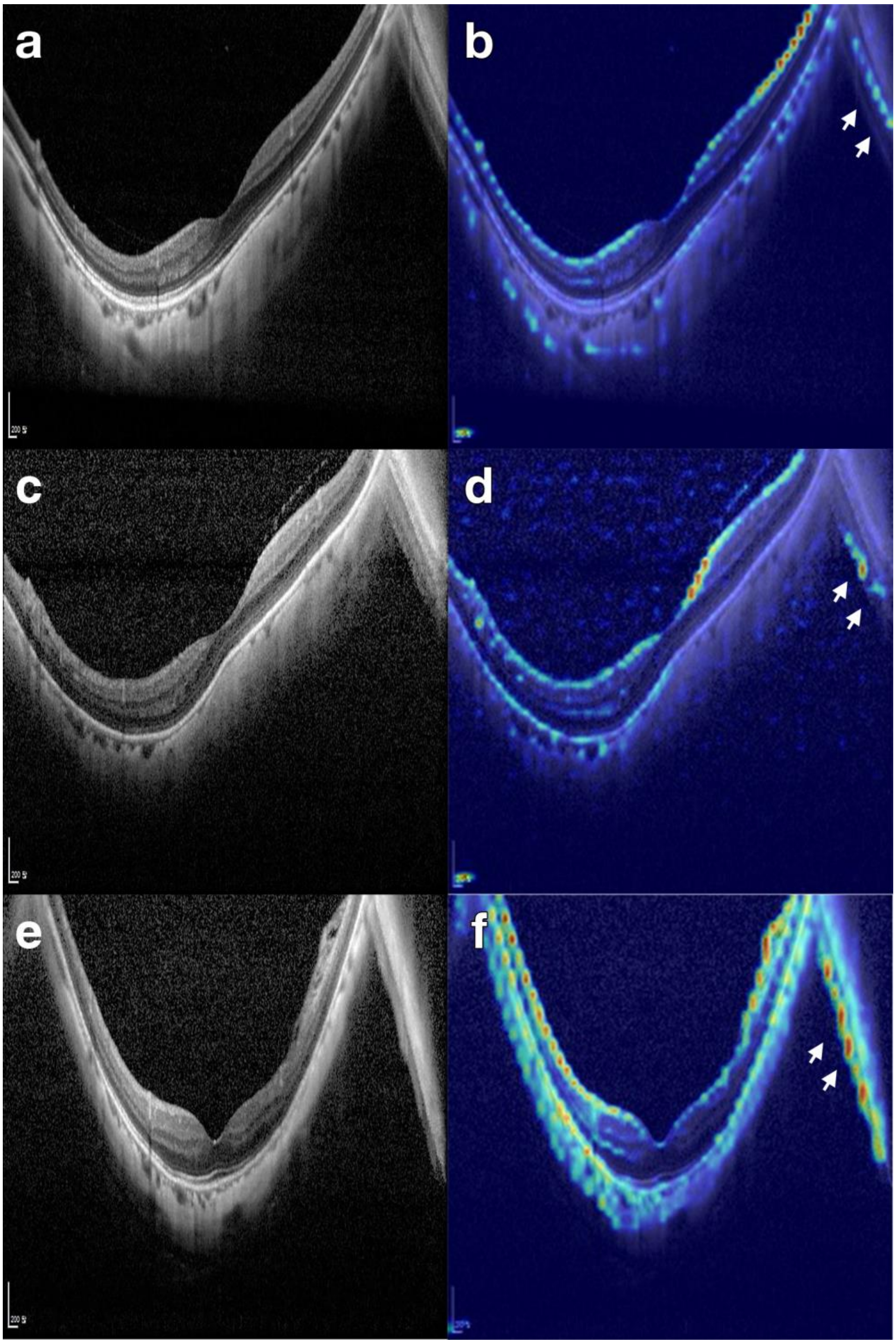

Supplementary Figure 1 Examples of the DL algorithm recognizing flipped images and highlighting the retinal nerve fiber layer of eyes with mirror-image artifacts. (**a, c, e**) Macular vertical OCT scans. (**b, d, f**) The same images as in panel (a, c, e) after highlighting by the deep learning algorithm. Due to the highly bowed posterior pole, a cross-sectional image could not be fully captured in the scan window and the image over the frame is flapped. Although the image quality was lower in the mirror image than in the original image, the deep learning algorithm noticed the flapped image and highlighted the retinal nerve fiber layer of the image (white arrows).

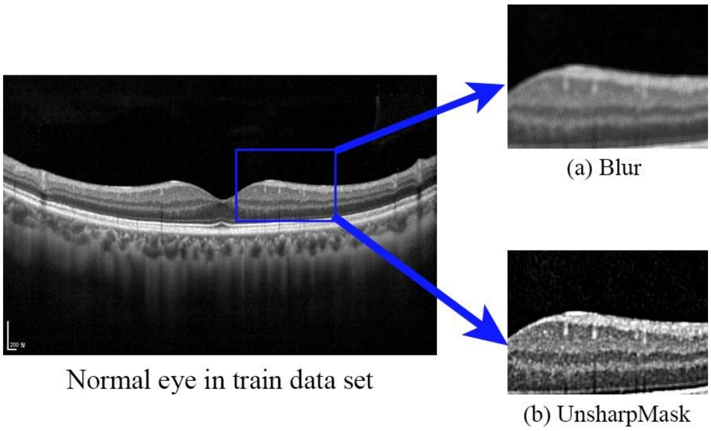

Supplementary Figure 2 Examples of augmentation of blur and unsharp mask phases.

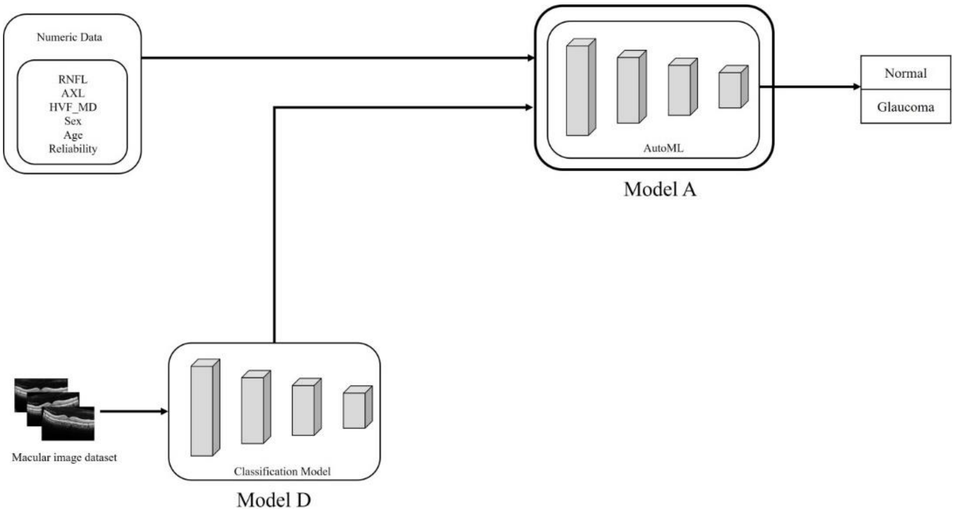

Supplementary Figure 3 Combined model with baseline demographic and ophthalmic characteristics.

Supplementary Table 1. AUC results of the deep learning system using each model to diagnose myopic glaucoma in the external test dataset, based on circumpapillary and macular vertical OCT scans.

	AUC (95% Confidence Interval)	
	**Circumpapillary OCT**	**Macular Vertical OCT**
DenseNet-121	0.580 (0.483–0.676)	0.709 (0.620–0.798)
VGG-13	0.535 (0.437–0.633)	0.859 (0.791–0.928)
ResNet-34	0.623 (0.483–0.676)	0.540 (0.442–0.638)
ResNet-101	0.723 (0.635–0.810)	0.684 (0.593–0.775)
EfficientNet-B0	0.865 (0.798–0.932)	0.982 (0.956–1.000)
EfficientNet-B1	0.985 (0.961–1.000)	0.984 (0.959–1.000)

Abbreviations: AUC, area under the receiver operating characteristic curve; OCT, optical coherence tomography.
